# Reproducible Semi-Automated Quantification of Vascularization in Bone Sections Using CD31 Immunohistochemistry and Trainable Weka Segmentation

**DOI:** 10.3390/jimaging12090409

**Published:** 2026-09-01

**Authors:** Nick Mattern, Holger Freischmidt, Matthias Schulte, Alma Aubert, Sanja Kalmus, Jan Makogon, Paul Alfred Grützner, Jonas Armbruster, Felix Lamadé-Dootz

**Affiliations:** BG Klinik Ludwigshafen, Department for Orthopaedics and Trauma Surgery, Heidelberg University, 67071 Ludwigshafen, Germany; holger.freischmidt@bgu-ludwigshafen.de (H.F.); matthias.schulte@bgu-ludwigshafen.de (M.S.); alma.aubert@bgu-ludwigshafen.de (A.A.); sanja.kalmus@bgu-ludwigshafen.de (S.K.); jan.makogon@stud.uni-heidelberg.de (J.M.); jonas.armbruster@bgu-ludwigshafen.de (J.A.)

**Keywords:** bone, CD31 immunohistochemistry, vascular histomorphometry, bone regeneration, bone defect model, angiogenesis, Trainable Weka Segmentation, Fiji/ImageJ, semi-automated image analysis, CD31-positive area fraction

## Abstract

Quantitative assessment of vascularization is important in bone regeneration research, but CD31-immunohistochemically stained sections are often evaluated manually or semi-quantitatively, limiting reproducibility and comparability. The aim of this study was to establish and validate a reproducible, open-source workflow for semi-automated quantification of CD31-positive area fraction in bone sections using Fiji/ImageJ and Trainable Weka Segmentation (TWS). CD31-immunohistochemically stained rat bone sections from defect/regenerating tissue, femur, tibia, and spine were analyzed. The workflow combined standardized image acquisition, predefined regions of interest, pixel-based TWS classification, extraction of the CD31-positive class, and CD31-positive area normalized to tissue area (CD31.Ar/T.Ar). Manual reference measurements were performed by two independent observers in repeated runs. Manual CD31.Ar/T.Ar measurements showed good retest reliability, with mean coefficients of variation (CV) of 7.17% and 6.84% for observer 1 and observer 2, respectively, and good interobserver agreement. Independently trained TWS classifiers produced highly stable CD31.Ar/T.Ar values, with an overall mean CV of 1.98%. Manual assessment required 3:17 ± 1:35 min per section, whereas the TWS-based workflow separated an initial classifier training step from rapid repeated analysis of larger image sets. This study provides a transparent, reproducible, and time-efficient open-source workflow for semi-automated quantification of CD31-positive vascular area fraction in bone sections and supports its use as a scalable method for vascular histomorphometry in preclinical bone regeneration research.

## 1. Introduction

Bone regeneration is a highly coordinated biological process that depends not only on osteogenic cells and the formation of mineralized matrix but also on adequate vascularization. Newly formed blood vessels provide oxygen, nutrients, systemic signals, and inflammatory, stromal, and osteogenic precursor cells to the regenerating tissue, thereby supporting bone repair [1,2]. In fracture healing and bone defect regeneration, the functional interaction between angiogenesis and osteogenesis is essential for successful tissue repair [3]. Specific vascular subtypes, especially CD31 (platelet endothelial cell adhesion molecule-1) positive vessels, have been associated with osteogenic niches and bone formation [4,5]. At the same time, recent studies indicate that vascular sprouting and osteoprogenitor invasion may also be spatially or temporally uncoupled in certain bone regeneration models [6]. These findings highlight the need for reproducible histological workflows that allow objective assessment of vascularization in bone repair models.

Preclinical and translational bone research commonly integrates multiple methods, including radiology, micro-computed tomography, histology, immunohistochemistry, gene expression analysis, and serum-based assays, to obtain a comprehensive understanding of bone repair. While radiological and microstructural techniques provide essential information on bone volume, mineralization, and architecture, histology remains indispensable for assessing cellular and tissue-level changes within their biological context [7]. Quantification of CD31-positive vascularization adds complementary information on the endothelial and angiogenic component of bone regeneration, which cannot be directly derived from mineralized tissue volume, architecture, or conventional morphological assessment alone. Bone histomorphometry enables the quantitative evaluation of bone architecture, bone formation, bone resorption, and cellular activity. It is highly relevant not only for systemic skeletal diseases such as osteoporosis and osteomalacia but also for experimental studies of fracture and defect healing [7,8]. The scientific community has provided standardized nomenclature, symbols, units, and methodological recommendations for the assessment of bone parameters according to the American Society for Bone and Mineral Research (ASBMR) [8,9]. These standards provide an important basis for comparable and reproducible histomorphometric analyses.

Despite this standardization, the quantitative analysis of histological bone sections remains time-consuming, observer-dependent, and methodologically heterogeneous in many research settings. This is particularly relevant for immunohistochemical vascular staining, where CD31-positive structures are often assessed manually, semi-quantitatively, or in selected image regions only. These approaches are sensitive to observer-dependent decisions, especially in sections with heterogeneous staining intensity, complex tissue architecture, small vascular profiles, background signal, cracks, folds, hematoma, or biomaterial remnants.

Advances in digital image analysis have promoted the development of both commercial and freely available software solutions for histological quantification. Commercial platforms, including MATLAB-based workflows and Visiopharm-based algorithms, have been successfully applied to automated or semi-automated bone histomorphometry [10,11]. However, the high cost of commercially available software limits its broad application, especially in smaller research groups, preclinical laboratories, and international collaborative projects. Open-source platforms offer a major advantage in this context because they are freely available, transparent, adaptable, and reproducible. ImageJ and Fiji have become widely used platforms for scientific image analysis [12]. Building on this environment, Trainable Weka Segmentation provides a user-friendly machine learning plugin for Fiji/ImageJ that enables pixel-based classification of microscopy images using user-defined training classes [13].

Several open-source approaches have already been established in the field of bone histomorphometry. Van’t Hof et al. developed Java-based ImageJ-compatible tools for the semi-automated assessment of bone resorption, osteoid parameters, and fluorochrome-labeled samples [14]. Malhan et al. adapted Trainable Weka Segmentation, ImageJ, and BoneJ to quantify parameters such as trabecular area, osteoid area, trabecular thickness, trabecular separation, and osteoclast activity across different histological stains and bone samples [15]. These studies demonstrate that open-source, user-friendly, and validated workflows can improve the efficiency and reproducibility of histomorphometric analyses. However, existing protocols primarily focus on classical bone parameters, osteoblast and osteoclast activity, osteoid, mineralization, or general tissue classes. A validated open-source workflow specifically designed for reproducible quantification of CD31-positive vascular signal area in bone sections has not yet been standardized. The novelty of the present study lies in the systematic validation of a freely accessible Fiji/ImageJ-based workflow specifically for CD31-positive vascular area quantification in bone tissue. In addition to establishing the analytical pipeline, we evaluated manual intra- and interobserver variability, technical repeatability, and the variability between independently trained TWS classifiers across different bone tissue architectures. This provides not only an open-source segmentation workflow but also a reproducibility framework for its application in preclinical bone vascularization studies.

The aim of this study was therefore to establish and validate a reproducible, semi-automated, and open-source workflow for quantifying CD31-positive area fraction in immunohistochemically stained bone sections.

## 2. Materials and Methods

### 2.1. Sample Material, Ethical Approval, and Histological Processing

For the establishment and validation of the semi-automated image analysis workflow, histological bone sections obtained from an approved animal experiment were used. No additional animals were used for the present methodological analysis. All underlying animal procedures were performed in accordance with institutional regulations and the applicable German and European animal welfare legislation. The animal experiment was approved by the State Investigation Office of Rhineland-Palatinate, Koblenz, Germany (Landesuntersuchungsamt Rheinland-Pfalz; approval no. 23 177-07/G 25-7-008).

After completion of the respective experimental endpoints, bone specimens were harvested and prepared for histological processing. Samples were fixed in 4% paraformaldehyde, rinsed in phosphate-buffered saline, and subsequently decalcified in EDTA solution. Decalcification was performed over several weeks depending on sample size, with regular exchange of the decalcification solution. After complete decalcification, specimens were rinsed and stored in 70% ethanol at 4–8 °C until further processing.

For paraffin embedding, samples were transferred into embedding cassettes and processed in an automated tissue processor (Leica Biosystems Nussloch GmbH, Nussloch, Germany) using a graded ethanol series, xylene clearing, and paraffin infiltration. Specimens were embedded in paraffin with standardized orientation to obtain reproducible section planes through the defect or regenerative region. Paraffin sections were then prepared and used for CD31 immunohistochemistry and subsequent digital image analysis.

Sample collection, decalcification, paraffin embedding, and immunohistochemical processing were performed according to standardized internal protocols to ensure reproducible tissue quality and a traceable basis for workflow development. The detailed decalcification and paraffin embedding procedure is summarized in Supplementary Table S1.

### 2.2. CD31 Immunohistochemistry

To visualize vascular structures, single immunohistochemical staining for CD31 was performed on paraffin-embedded rat bone sections. CD31, also known as platelet endothelial cell adhesion molecule-1 (PECAM-1), was used as an endothelial marker to identify blood vessels and capillaries within the defect or regenerating tissue. Each staining run included a negative control without primary antibody.

Before staining, freshly cut paraffin sections were incubated in a drying oven at 70 °C for at least 20 min. Sections were then deparaffinized and rehydrated using xylene (Carl Roth GmbH + Co. KG, Karlsruhe, Germany), a descending ethanol series (70%, 96%, 100%; Carl Roth GmbH + Co. KG, Karlsruhe, Germany), and distilled water. After rehydration, sections were pre-rinsed in distilled water for 5 min.

Antigen retrieval was performed in epitope retrieval solution (ERS; IHC WORLD, Ellicott City, MD, USA) for 60 min at 65 °C. Sections were then allowed to cool for 20 min at room temperature and washed three times for 5 min each in Tris-buffered saline (TBS), pH 7.6 (Carl Roth GmbH + Co. KG, Karlsruhe, Germany). Tissue sections were encircled using a hydrophobic PAP pen (ImmEdge Pen; Vector Laboratories Inc., Newark, NJ, USA). To reduce endogenous enzymatic activity, sections were incubated with 3% hydrogen peroxide in TBS (Carl Roth GmbH + Co. KG, Karlsruhe, Germany) for 20 min. This was followed by three washing steps for 5 min each in TBS containing Tween-20 (TBST; Carl Roth GmbH + Co. KG, Karlsruhe, Germany). To block nonspecific binding, sections were incubated with 2.5% horse serum (Vector Laboratories Inc., Newark, NJ, USA) for 30 min. Excess serum was carefully removed without allowing the sections to dry. The primary antibody against CD31, rabbit anti-CD31 (EPR17259; ab182981; abcam, Cambridge, UK), was diluted 1:1000 in 1% bovine serum albumin in TBS (BSA/TBS; Carl Roth GmbH + Co. KG, Karlsruhe, Germany). Sections were incubated with the primary antibody overnight at 4 °C in a humidified chamber.

On the following day, sections were washed three times for 5 min each in TBST (Carl Roth GmbH + Co. KG, Karlsruhe, Germany). Detection was performed for 30 min at room temperature using an alkaline phosphatase-conjugated anti-rabbit polymer, ImmPRESS-AP anti-rabbit IgG, MP-5401 (Vector Laboratories Inc., Newark, NJ, USA). Sections were then washed again three times for 5 min each in TBST (Carl Roth GmbH + Co. KG, Karlsruhe, Germany). The chromogenic reaction was developed using the Vector Red Substrate Kit SK-5105 (Vector Laboratories Inc., Newark, NJ, USA) supplemented with levamisole (Vector Laboratories Inc., Newark, NJ, USA) for 10–15 min and monitored microscopically. Levamisole was added to reduce endogenous alkaline phosphatase activity. The reaction was stopped by rinsing the sections in distilled water.

For counterstaining, sections were incubated for 30 s with Instant Hematoxylin (Sigma-Aldrich Chemie GmbH, Taufkirchen, Germany). Sections were then rinsed in distilled water for 1 min, dehydrated through 70% ethanol, 96% ethanol, and 100% ethanol, cleared in xylene, and mounted using RotiHistokitt (Carl Roth GmbH + Co. KG, Karlsruhe, Germany).

For negative controls, the same protocol was applied, but the primary antibody was replaced by the corresponding antibody diluent, 1% BSA/TBS (Carl Roth GmbH + Co. KG, Karlsruhe, Germany). Only staining runs showing specific CD31 staining of vascular structures and no relevant nonspecific signal formation in the negative control were included in the subsequent digital image analysis.

To optimize nuclear counterstaining while preserving clear contrast to the CD31-positive Vector Red signal, different Instant Hematoxylin incubation times were tested before finalizing the staining protocol. Sections were counterstained for 10 s, 20 s, 30 s, 45 s, 60 s, 2 min, and 5 min. An incubation time of 30 s was selected for the final protocol, as it provided sufficient tissue and nuclear contrast while maintaining clear visual separation from the CD31-positive vascular signal. Shorter incubation times resulted in weaker tissue contrast, whereas longer incubation times increased the intensity of the counterstain and reduced the visual contrast between the background tissue and CD31-positive structures. Representative images of the optimization are shown in Supplementary Figure S1.

### 2.3. Image Acquisition

Digital images were acquired using a Zeiss microscopy system (Carl Zeiss Microscopy GmbH, Jena, Germany), consisting of a light microscope (Axio Imager M2; Carl Zeiss Microscopy GmbH, Jena, Germany), a digital camera (AxioCam MRc; Carl Zeiss Microscopy GmbH, Jena, Germany), and the corresponding image acquisition software (AxioVision 4.8; Carl Zeiss Microscopy GmbH, Jena, Germany). The sections were imaged at 50×, 100×, 200×, and 400× magnification. The calibrated image resolution was 2.04 µm/pixel at 5× magnification, 1.02 µm/pixel at 10× magnification, 0.51 µm/pixel at 20× magnification, and 0.26 µm/pixel at 40× magnification. Images acquired at 10× magnification were primarily used for overview analysis and for defining larger regions of interest, whereas 20× images were used for more detailed assessment of smaller vascular structures, segmentation quality, and manual reference measurements. Depending on the respective validation step, 5× and 40× images were additionally used for low-magnification overview documentation and high-magnification quality control, respectively. Within each staining run, images were acquired using constant illumination, white balance, and exposure settings to minimize acquisition-related variation during subsequent digital image analysis.

### 2.4. Software and Semi-Automated Image Analysis Workflow

Digital image analysis was performed using Fiji/ImageJ (ImageJ version 1.54p; National Institutes of Health, Bethesda, MD, USA). The workflow is based on Java-compatible Fiji/ImageJ functions and can be executed on Windows, macOS, or Linux, provided that a compatible Java environment is available.

Trainable Weka Segmentation was used for semi-automated pixel classification of CD31-immunohistochemically stained sections. User-defined training regions were used to classify CD31-positive vascular signal, counterstained/background tissue, and relevant artifacts or empty background. After classifier training, the classified output image was generated using the “Create Result” function. The CD31-positive class was isolated by thresholding and converted into a binary mask. The total CD31-positive area was then quantified using the “Analyze Particles” function in Fiji/ImageJ. The final automated endpoint was CD31.Ar/T.Ar (%), calculated from the total CD31-positive area divided by the predefined ROI area.

### 2.5. Reproducibility and Validation

To evaluate the reproducibility and validity of the semi-automated workflow, repeated TWS-based measurements were compared with manual measurements performed in Fiji/ImageJ. A defined validation dataset of representative histological bone sections was selected for this purpose. The dataset was designed to include different staining intensities, vascular densities, tissue structures (spine, femur, femoral defect, and tibia), and common histological artifacts to assess the robustness of the workflow under realistic analysis conditions.

For technical repeatability of the semi-automated analysis, the same section or predefined region of interest was analyzed repeatedly using the TWS-based workflow. Each measurement was repeated in independent runs. Identical ROI boundaries were used to capture variability related to segmentation and downstream mask analysis. For comparison with the TWS-based workflow, the same sections were evaluated manually in Fiji/ImageJ. One investigator manually measured the predefined CD31-positive vascular structures in repeated independent runs. A second independent investigator performed the same manual analysis on the identical image dataset.

### 2.6. Region of Interest Definition and Image Calibration

**Figure 1:**
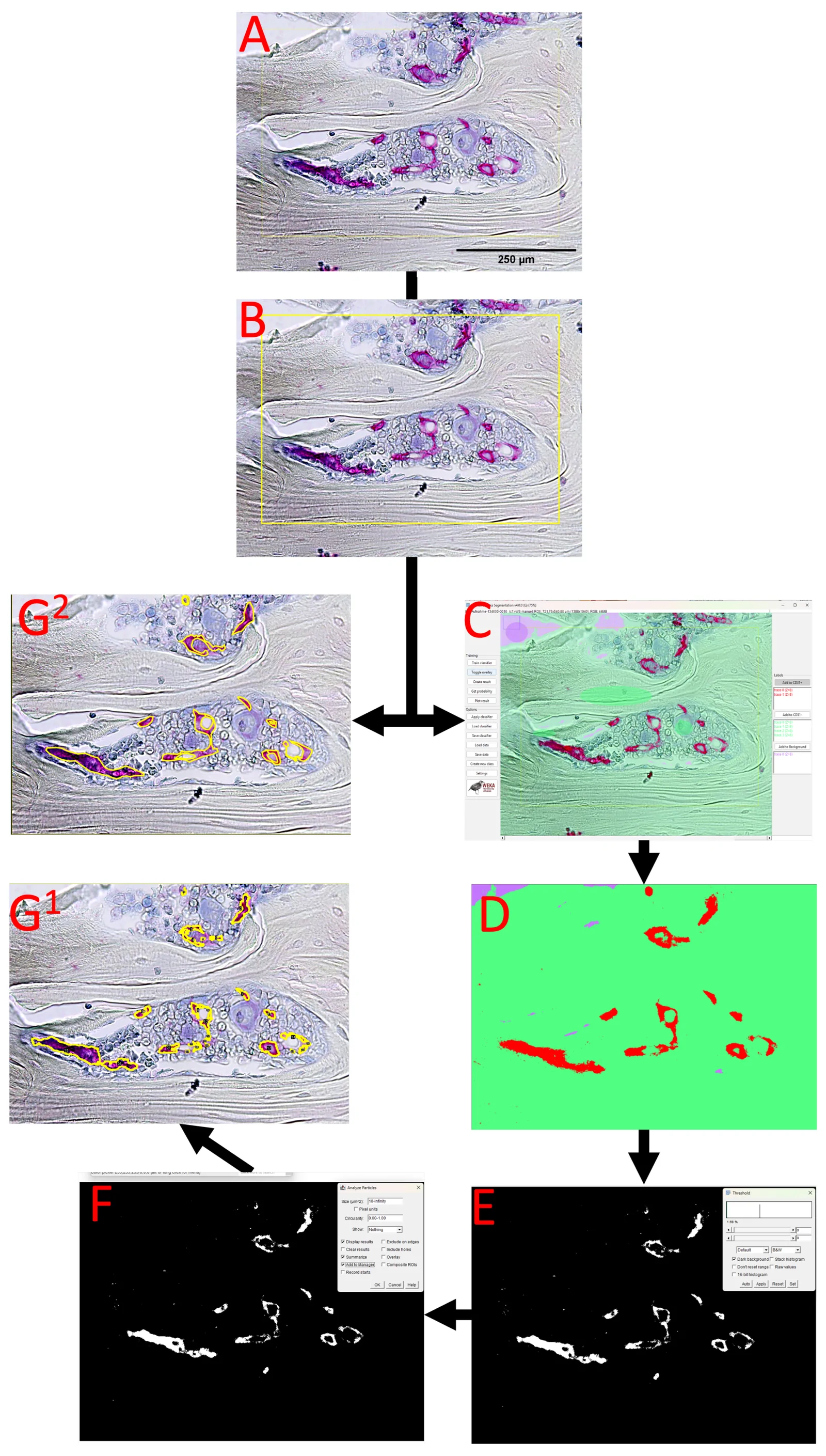
Representative workflow illustrating ROI definition and visual performance of Trainable Weka Segmentation for CD31-positive area detection. (**A**) Representative CD31-immunohistochemically stained bone section with scale bar. The scale bar shown in (**A**) applies to all panels. (**B**) Illustrative definition of a region of interest (ROI) to demonstrate the obligatory ROI-selection step. (**C**) Example of the Trainable Weka Segmentation interface after classifier training and visual inspection of the predicted CD31-positive class. (**D**) Resulting classified image showing CD31-positive signal (red) separated from background tissue (green/purple). (**E**) Thresholding of the CD31-positive class to generate a binary mask (CD 31 positive is white; background is black). (**F**) Subsequent particle analysis of the binary mask in Fiji/ImageJ (CD 31 positive is white; background is black). (**G^1^**) Overlay of the TWS-derived segmentation result onto the original histological image. The outlined regions (yellow) correspond to CD31-positive structures identified from the TWS-generated binary mask and subsequent particle analysis. (**G^2^**) Overlay of the manual delineation (yellow) of CD31-positive vascular structures on the same histological image, allowing direct visual comparison of automated and observer-defined vessel boundaries. A detailed step-by-step description of the complete measurement workflow is provided in Supplementary Protocol S1.

Regions of interest (ROIs) were defined in Fiji/ImageJ according to the anatomical structure of the respective section and the intended validation region. The ROI shown in Figure 1 was selected solely as an illustrative example to demonstrate the obligatory ROI-definition step and does not represent a standardized anatomical target area or the exclusion of a preparation artifact. In hypothesis-driven applications of the workflow, the anatomical ROI should instead be predefined a priori according to the specific experimental design, biological question, and reproducible anatomical landmarks. Because the workflow is intended to be adaptable to different bone tissues and experimental designs, no fixed universal ROI geometry was imposed. Instead, each ROI was predefined before quantitative analysis, saved in the ROI Manager, and applied identically to the manual reference measurements and the TWS-based analysis.

For the validation dataset, ROIs included representative regions from defect/regenerating tissue, femur, tibia, and spine sections. These regions were used to assess the workflow across different tissue architectures and staining patterns rather than to define standardized anatomical compartments. When present, obvious preparation artifacts or tissue outside the predefined target ROI were excluded before quantitative analysis. These general exclusion criteria are independent of the illustrative ROI shown in Figure 1. Image calibration was performed using the microscope-specific scale information. For the validation images, the calibrated resolution was 1.02 µm/pixel at 10× magnification, 0.51 µm/pixel at 20× magnification, and 0.255 µm/pixel at 40× magnification. ROI area was measured separately from the saved ROI and used as the denominator for calculation of CD31.Ar/T.Ar.

### 2.7. Trainable Weka Segmentation and Classifier Application

Semi-automated segmentation was performed using Trainable Weka Segmentation in Fiji/ImageJ. User-defined training regions were annotated to separate CD31-positive vascular signal from counterstained tissue, empty background, and relevant artifact-related signal. Training regions were selected to include different staining intensities, tissue textures, and common histological artifacts observed in the validation dataset.

After training, the classifier was applied to the validation images, and a classified output image was generated using the “Create Result” function. The classified TWS output contained discrete pixel values representing the respective classifier-defined classes. Therefore, no additional intensity-based threshold for CD31 positivity was empirically selected. Thresholding was used only to isolate the discrete pixel value assigned to the CD31-positive class and to convert these pixels into a binary mask for subsequent area measurement. The resulting binary CD31-positive mask was restricted to the predefined ROI by clearing all image areas outside the saved ROI. The binary mask was then analyzed using the “Analyze Particles” function in Fiji/ImageJ to obtain the total CD31-positive area. Three independently trained classifiers were generated by three different investigators from the department, including two postdoctoral researchers and one doctoral researcher. Classifier training was performed iteratively by adding representative annotations from CD31-positive signal, counterstained tissue, background, and ambiguous transition regions until visual inspection showed reliable separation of the different classes. Particular attention was paid to subtle color contrasts and transition zones between the blue hematoxylin-counterstained tissue and the red CD31-positive chromogenic signal. The three independently generated classifiers were subsequently applied to the same image set to assess classifier-dependent variability.

### 2.8. Quantitative Endpoint Definition

The primary quantitative endpoint of the automated workflow was CD31-positive area fraction, expressed as CD31.Ar/T.Ar (%). CD31.Ar/T.Ar was calculated from the total CD31-positive area measured in the raw TWS-derived binary mask and normalized to the corresponding ROI area: CD31.Ar/T.Ar (%) = total CD31-positive area/ROI area × 100.

### 2.9. Manual Reference Measurements, Statistical Analysis, and Runtime Assessment

Manual reference measurements were performed in Fiji/ImageJ using the same image data and predefined ROIs as the TWS-based workflow. CD31-positive vascular signal areas were manually delineated according to standardized criteria. Each sample was measured three times by one observer to assess retest reliability. A second independent observer repeated the same manual analysis on the identical image dataset to assess interobserver agreement.

For each individual measurement, CD31.Ar/T.Ar (%) was calculated as the total manually delineated CD31-positive area divided by the corresponding predefined tissue or ROI area × 100. For observer-specific retest analysis, the three repeated measurements of each sample were used to calculate a sample-specific mean, standard deviation (SD), and coefficient of variation (CV; SD/mean × 100). These sample-specific values were subsequently summarized across the complete validation dataset. Accordingly, the values reported in Table 1 represent the mean of the sample-specific means, the mean of the sample-specific SDs, the mean of the sample-specific CVs, and the SD of the sample-specific CVs. For interobserver analysis, the three repeated measurements were first averaged within each observer for each sample, and interobserver variability was subsequently calculated between the two observer-specific sample means.

TWS-based measurements were compared with the manual reference measurements. For automated analysis, three independently trained TWS classifiers were applied to the same image set in repeated runs to evaluate technical repeatability and classifier-dependent variability. Mean values, standard deviations, and coefficients of variation (CV) were calculated for repeated measurements. Interobserver agreement and method agreement were assessed using Pearson correlation coefficients and intraclass correlation coefficients where applicable. For the comparison between manual and TWS-based measurements, repeated measurements were first averaged within each sample, thereby avoiding the treatment of repeated technical measurements as independent observations. The manual value for each sample was calculated from the observer-specific sample means, whereas the TWS value was calculated from the three independently trained classifier-specific sample means, yielding one paired manual and TWS value for each of the 12 samples. Normality of the paired differences was assessed using the Shapiro–Wilk test. As the paired differences showed no evidence of deviation from normality, manual and TWS-derived CD31.Ar/T.Ar values were compared using a two-sided paired *t*-test. Statistical significance was defined as *p* < 0.05.

The time required for manual CD31.Ar/T.Ar measurement was recorded for both observers during one complete measurement run. Runtime was analyzed per section and summarized overall and by anatomical region.

### 2.10. Data, Code, and Protocol Availability

The detailed step-by-step Fiji/ImageJ protocol is available as Supplementary Material on GitHub. Furthermore, the customized BeanShell script for fully automated analyses is provided at https://github.com/Nick-Mattern/tws-area-makro (accessed on 2 June 2026). Because Trainable Weka Segmentation classifiers depend on staining intensity, imaging conditions, and sample characteristics, the provided classifier workflow is intended as a reproducible framework and should be retrained or adapted using representative images from each individual dataset.

## 3. Results

### 3.1. Establishment of a Semi-Automated TWS-Based Workflow for CD31-Positive Area Quantification

A semi-automated image analysis workflow was established to quantify CD31-positive vascular signal in histological bone sections. The resulting TWS-based segmentation enabled reproducible extraction and quantification of CD31-positive area within predefined ROIs.

Visual inspection of the classified images and binary masks showed that the TWS-based approach reliably detected CD31-positive signal areas in different bone section types. The workflow was applicable to sections from defect/regenerating tissue, femur, tibia, and spine samples, indicating that the method can be applied across different bone tissue architectures and staining intensities. Representative steps of the workflow, including the original image, ROI definition, classified TWS output, thresholded CD31 mask, and particle analysis output, are shown in Figure 1.

### 3.2. Manual Reference Measurements Showed Good Retest and Interobserver Reliability

Repeated measurements by the same observer showed low intraobserver variability. The mean coefficient of variation for CD31.Ar/T.Ar was 7.17% for observer 1 and 6.84% for observer 2. These values indicate that manual CD31.Ar/T.Ar assessment was reproducible when identical ROIs and standardized measurement criteria were used (Table 1).

Interobserver comparison between both manual observers demonstrated good agreement for CD31.Ar/T.Ar. The Pearson correlation coefficient was 0.906, and the intraclass correlation coefficient was 0.869. The mean absolute interobserver difference was 1.17 ± 0.97 percentage points. When interobserver variability was calculated from the two observer-specific sample means, the mean interobserver SD was 0.83 and the mean interobserver CV was 14.24% (Table 1). These results indicate that the manual CD31.Ar/T.Ar measurements were sufficiently reproducible to serve as a reference for evaluating the semi-automated TWS-based workflow.

### 3.3. TWS-Based CD31 Area Fraction Was Stable Across Repeated Measurements and Independently Trained Classifiers

To evaluate the robustness of the semi-automated workflow, three independently trained TWS classifiers were applied to the same image set. Each classifier was applied in three repeated runs. The automated endpoint was CD31.Ar/T.Ar derived from the raw TWS-generated CD31-positive mask. For all automated measurements, CD31.Ar/T.Ar was calculated from the total CD31-positive area measured by particle analysis and normalized to the final ROI area.

Repeated application of the same trained classifier produced identical CD31.Ar/T.Ar values (Table 2), confirming deterministic technical repeatability of the analysis pipeline. Comparison between independently trained classifiers showed highly similar CD31.Ar/T.Ar values across the validation dataset. Mean CD31.Ar/T.Ar was 6.06% for classifier 1, 6.02% for classifier 2, and 6.07% for classifier 3. Across all classifiers and runs, mean CD31.Ar/T.Ar was 6.05%, with a mean SD of 0.09 and a mean CV of 1.98% (Table 2). These findings indicate that automated extraction of CD31-positive area fraction is highly stable when classifiers are trained consistently and applied to standardized ROIs.

### 3.4. Manual and TWS-Based Measurements Quantified Related but Non-Identical Endpoints

Manual and TWS-based CD31.Ar/T.Ar measurements were compared to evaluate the relationship between observer-defined CD31-positive area measurements and the semi-automated TWS-derived CD31-positive signal area fraction. Manual values were calculated as the mean of both observers, whereas TWS values were calculated as the mean of the three independently trained classifiers.

**Figure 2:**
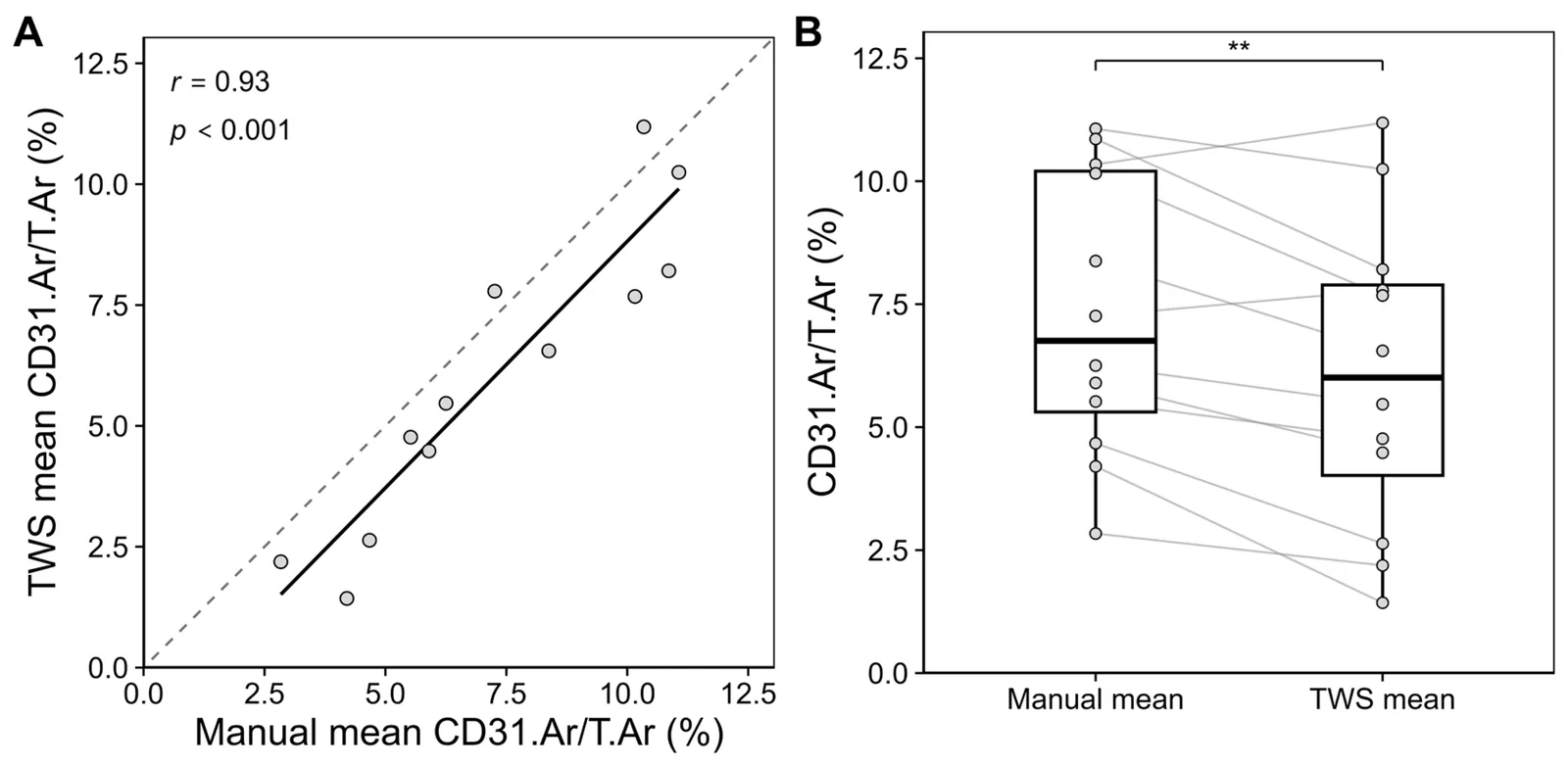
Comparison of manual and TWS-derived CD31.Ar/T.Ar measurements: (**A**) Correlation between manual and TWS-derived CD31.Ar/T.Ar measurements. Scatterplot of mean manual versus mean TWS CD31-positive area fraction per sample, with the solid line indicating the linear regression and the dashed line the line of identity. Pearson’s correlation coefficient (r = 0.93, *p* < 0.001) demonstrates a strong positive association between manual and TWS measurements. (**B**) Paired comparison of manual mean measurements and TWS-derived measurements for CD31-positive area fraction. Manual values represent the mean of both observers; TWS values represent the mean of three independently trained classifiers. Manual and TWS-derived values were compared across the 12 paired samples using a two-sided paired *t*-test. Star coding: ** *p* < 0.01.

TWS-derived CD31.Ar/T.Ar values showed a positive relationship with manual measurements across samples. The Pearson correlation coefficient between manual and TWS-derived CD31.Ar/T.Ar was 0.93. Across the full validation set, the mean bias between TWS and manual measurements was −1.24 percentage points, with a mean absolute difference of 1.46 percentage points. The paired comparison using a two-sided paired Student’s *t*-test indicated that TWS-derived CD31.Ar/T.Ar values were significantly lower than the corresponding manual measurements (*p* < 0.01; Figure 2B). Individual samples showed larger deviations, particularly in regions with complex tissue architecture or heterogeneous staining.

Region-wise comparison showed that the workflow performed across different bone tissue types, including defect/regenerating tissue, femur, tibia, and spine (Table 3). Representative original images, corresponding TWS-derived binary masks, and segmentation overlays from samples included in the quantitative validation are provided in Supplementary Figure S2, allowing visual assessment of the segmentation results underlying the measurements reported in Table 3.

### 3.5. Time Required for Manual and TWS-Based Analysis

The time required for manual CD31.Ar/T.Ar assessment was recorded for both observers during one complete measurement run. Across all analyzed sections and both observers, manual assessment required 3:17 ± 1:35 min per section. The mean analysis time was comparable between observers, with 3:15 ± 1:35 min per section for observer 1 and 3:19 ± 1:36 min per section for observer 2.

Analysis time varied depending on anatomical region and vascular complexity. Defect/regenerate sections required the longest manual analysis time, with 5:12 ± 0:31 min per section. Femur sections required 3:38 ± 0:16 min, spine sections 3:13 ± 0:52 min, and tibia sections 1:05 ± 0:24 min per section. This indicates that manual analysis time was strongly influenced by the amount and complexity of CD31-positive signal and by the effort required for manual delineation within the predefined ROI.

The TWS-based workflow differs fundamentally from manual measurement because it separates an initial classifier training step from subsequent repeated image analysis. The first use of the workflow requires training and visual quality control of the classifier. Once a representative classifier has been trained and saved, subsequent analysis no longer requires manual delineation of all CD31-positive structures.

For images of the size used in the present validation dataset, generation and quantitative processing of the TWS result required approximately 2 min per image on the least performant workstation used in our laboratory, which was equipped with 64 GB of RAM. This value should be regarded as an approximate dataset- and hardware-specific runtime rather than a generally applicable processing time. TWS processing time increases with image dimensions and total pixel number and is also influenced by the available system memory, the amount of memory allocated to Fiji/ImageJ, CPU performance, and the complexity of the image features used for classification. Consequently, substantially larger whole-section or high-resolution images may require considerably longer processing times.

Despite this hardware-dependent computational time, the trained classifier allows the subsequent segmentation and quantitative analysis to proceed without repeated manual delineation of individual vascular structures, which becomes particularly advantageous in larger datasets.

## 4. Discussion

### 4.1. Comparison of Inter- and Intrarater Variability of TWS and Manual Assessment

Manual histomorphometric assessment of CD31-positive vascular structures showed a relevant degree of variability within observers. Intrarater variability during manual measurements was consistently higher than that observed with the TWS-based workflow, indicating that the classifier provides a more consistent and reliable read-out for vascularization metrics. Interrater coefficients of variation in the TWS workflow were 1.98%, which is in line with or below previously reported values for semi-automated open-source histomorphometric methods [15,16]. Van’t Hof et al. reported similarly low variability in bone histomorphometry [14]. Tong et al. have documented interobserver coefficients of variation of up to approximately 16% for selected bone parameters, particularly for osteoid-related measurements characterized by diffuse boundaries and heterogeneous staining [17]. In contrast, the parameters assessed in the present work are derived from sharply defined CD31-positive endothelial profiles, which likely contributes to the lower interrater variability observed.

When directly compared with fully manual segmentation, the advantages of the TWS-based workflow are evident both in terms of reproducibility and time efficiency. Manual assessments exhibited noticeably higher intrarater variability, reflecting the susceptibility of visual delineation to subjectivity, observer fatigue, and subtle changes in inclusion criteria over time. A likely explanation is that human observers tend to inconsistently include or exclude small, faint, or borderline regions, thereby introducing additional noise into the measurements. By contrast, the trained classifier applies a fixed set of learned features to all images, ensuring consistent identification of CD31-positive areas and reducing the risk of inadvertently including non-vascular structures or missing small vessels. This robustness is particularly relevant in larger datasets and multicenter studies, where standardization across observers and time points is critical.

In addition, TWS analyzes image data at the pixel level with a spatial resolution that would be impractical to reproduce manually, as manual observers would need to zoom in extensively to approximate the same level of boundary precision.

Consequently, the margin for error in manually delineated vessel areas is inherently higher than in a classifier-based approach that consistently applies the same learned decision rules across all images. Manual tracing has a finite spatial precision and is susceptible to small deviations in cursor positioning and boundary interpretation; computer-assisted tracing approaches have previously been shown to reduce spatial tracing deviations and observer variability compared with fully manual delineation [18]. This effect is particularly relevant for small vascular profiles, where even a displacement of only one or a few pixels along the vessel boundary can substantially affect the calculated area. Interestingly, Malhan et al. similarly reported higher manually determined area fractions compared with TWS-based measurements [15]. Thus, the significantly lower CD31.Ar/T.Ar values obtained with the TWS approach compared with manual assessment (Figure 2 and Table 3) may partly result from slightly broader delineation of vessel boundaries during manual tracing. However, we acknowledge that this is not the only possible explanation. TWS may conversely underestimate weakly stained capillaries, small or discontinuous vascular fragments, or CD31-positive signals within heterogeneous or hematoma-rich tissue. As no independent ground truth was available, the observed offset should therefore be interpreted as a method-dependent difference in boundary delineation and signal classification rather than as evidence that either method represents the absolute true vascular area. For the same reason, no calibration coefficient was applied to force TWS-derived measurements onto the manual scale, as this would require assuming manual delineation to represent an unbiased reference standard.

Time requirements further underscore the practical benefit of the semi-automated pipeline, as has previously been described [19]. Training the TWS classifier required approximately 15–20 min and needed to be performed only once per staining batch or experimental setup.

For images corresponding to the dimensions used in the present validation dataset, computational processing with the trained classifier required approximately 2 min per image on the least performant workstation tested (64 GB RAM). However, this runtime cannot be generalized, as TWS processing time scales with image size and pixel number and depends on available and Fiji/ImageJ-allocated memory, CPU performance, and the complexity of the selected image features. Importantly, computational processing does not require continuous user interaction once the classifier and analysis pipeline have been established. Larger image batches can therefore be processed unattended, for example, overnight, substantially reducing the amount of active operator time required. Thus, the principal time-saving advantage of the workflow should not be interpreted as a fixed computational runtime but rather as a reduction in repeated observer-dependent manual delineation and active user involvement once a classifier has been established.

### 4.2. Choice of CD31 as Endothelial Marker

An important methodological aspect of this work is the selection of CD31 as the endothelial marker for classifier training. The histological samples analyzed originated from a rat non-union model treated with either a ceramic bone substitute or autologous bone graft, in which the primary objectives were to quantify angiogenesis, vessel density, and vessel area within the defect region. CD31 (PECAM-1) is widely recognized as a standard endothelial marker for the assessment of angiogenesis, as it labels a broad spectrum of vessels irrespective of size or maturation status and is routinely used in both experimental and clinical histopathology [20]. Alternative endothelial or vascular markers such as CD105 (Endoglin), endomucin, and α-smooth muscle actin (α-SMA) have more specialized expression profiles and are commonly used in tumor angiogenesis research [4,5,21,22]. CD105 and endomucin preferentially label highly proliferative and strongly angiogenic endothelial cells and may therefore underrepresent quiescent or less active vessels, whereas α-SMA predominantly stains vascular smooth muscle cells and perivascular mural cells, emphasizing vessel maturity and remodeling rather than total vessel density. For the present application, which required comprehensive quantification of the entire vascular network within the defect, CD31 thus provided the most suitable compromise, capturing both immature and mature vessels within a single staining protocol. Although the classifier was specifically trained on CD31-stained sections, the underlying workflow is conceptually transferable to other markers and multiplexed staining strategies.

While CD31 was selected in the present study to provide a broadly applicable measure of endothelial vascular area, vascularization is a multidimensional process that cannot be completely characterized by a single immunohistochemical marker or area-based endpoint. Depending on the biological question, complementary markers may provide additional information on specific vascular phenotypes.

Accordingly, the present workflow should primarily be regarded as a standardized approach for quantifying a defined immunohistochemical vascular signal rather than as a comprehensive assessment of all aspects of bone vascularization. Provided that staining characteristics are sufficiently distinct and representative training data are available, the underlying TWS-based segmentation principle may also be adapted to other vascular markers or combinations of markers, as TWS-based workflows have previously been applied to different histological and immunohistochemical signals [15]. However, such applications would require marker-specific classifier training and separate validation.

### 4.3. Limitations

Despite its advantages, the TWS-based approach also has several limitations. First, although the use of a predefined training set and standardized features increases reproducibility, the outcome of the classifier still depends on the way it is initially trained. Different users may select slightly different training regions or class balances, which can lead to subtle discrepancies between independently trained classifiers when analyzing CD31-stained images.

In the present validation, the three classifiers were independently generated by two postdoctoral researchers and one doctoral researcher. The doctoral researcher had received specific training in the TWS workflow from the more experienced postdoctoral researchers prior to independent classifier generation. Therefore, the low inter-classifier variability observed in this study (CV 1.98%) reflects reproducibility under harmonized training conditions and should not necessarily be extrapolated to completely inexperienced users or laboratories without prior methodological standardization.

For optimal reproducibility across laboratories, it is therefore advisable to share reference training sets, provide detailed training guidelines, or distribute a pre-trained classifier together with the workflow.

Second, machine learning-based pixel classification is inherently constrained by the visual similarity between the true signal and background. In CD31-stained sections, non-specific chromogen deposition, uneven counterstaining, or tissue folds can generate regions whose pixel intensities and textures closely resemble those of true vessels, causing the classifier to erroneously label such areas as vascular structures. To mitigate this effect, optimization of the immunohistochemical staining protocol is essential to achieve high contrast between the CD31-positive endothelium and the surrounding tissue, thereby maximizing separability of vessel and background classes. In the presence of large artifacts, such as tissue folds, section borders, or strongly over-stained regions, manual exclusion prior to classifier training and inference may be necessary. In practice, these regions can be outlined with the polygon selection tool in Fiji and removed using the “Edit → Clear” function. This preprocessing step ensures that the classifier is trained on representative tissue only and reduces the likelihood that obvious artifacts propagate into the final segmentation and quantitative read-outs.

More generally, segmentation performance depends on the consistency of staining, tissue preparation, and image acquisition. Moderate variability in staining intensity or tissue appearance can be accommodated when representative examples are included during classifier training. However, substantial changes in chromogen intensity, counterstaining, image illumination, or sample quality may alter the visual features used for classification and can therefore reduce segmentation performance. When applying the workflow to a new staining batch, imaging setup, or substantially different tissue preparation, representative images should be visually inspected, and the classifier should be retrained or adapted if necessary. Consistent sample preparation, standardized image acquisition, and routine visual quality control therefore remain important prerequisites for reliable quantitative analysis.

## 5. Conclusions

The semi-automated histomorphometric analysis based on TWS proved to be straightforward to implement, reproducible, and substantially faster than conventional manual evaluation of CD31-positive vascular areas. Given its open-source implementation, high reproducibility, and scalability, this TWS-based workflow represents a valuable tool for future quantitative analyses of angiogenesis in preclinical bone healing studies and related imaging applications.

## Figures and Tables

**Table 1:** Reproducibility of manual CD31-positive area fraction measurements. CD31.Ar/T.Ar (%) was calculated for each measurement as the total manually delineated CD31-positive area divided by the corresponding predefined tissue or ROI area × 100. Each sample was measured three times by each observer. For observer-specific retest reliability, a mean, SD, and CV (SD/mean × 100) were calculated from the three repeated measurements of each sample. Values shown in the table represent the mean of the sample-specific means (“Mean”), the mean of the sample-specific SDs (“Mean SD”), the mean of the sample-specific CVs (“Mean CV”), and the standard deviation of the sample-specific CVs (“CV SD”) across all samples. For interobserver assessment, the three repeated measurements were first averaged within each observer for each sample, and variability was subsequently calculated between the two observer-specific sample means. CD31.Ar = CD31-positive area; T.Ar = predefined tissue or ROI area.

Method	Parameter	Mean	Mean SD	Mean CV (%)	CV SD (%)
Observer 1	CD31.Ar/T.Ar (%)	7.06	0.46	7.17	7.72
Observer 2	CD31.Ar/T.Ar (%)	7.52	0.51	6.84	4.57
Interobserver	CD31.Ar/T.Ar (%)	7.29	0.83	14.24	15.05

**Table 2:** Independently trained TWS classifiers were applied in repeated runs to the same image set. Values represent the mean of sample means, mean standard deviation, mean coefficient of variation (CV), and standard deviation of the coefficient of variation (CV SD) derived from the raw TWS-generated CD31-positive mask and normalized to the final ROI area. CD31.Ar/T.Ar = CD31-positive area/tissue or ROI area (%).

Method	Parameter	Mean	Mean SD	Mean CV (%)
TWS classifier 1	CD31.Ar/T.Ar (%)	6.06	0.00	0.00
TWS classifier 2	CD31.Ar/T.Ar (%)	6.02	0.00	0.00
TWS classifier 3	CD31.Ar/T.Ar (%)	6.07	0.00	0.00
All TWS classifiers	CD31.Ar/T.Ar (%)	6.05	0.09	1.98

**Table 3:** Comparison of manual and TWS-derived CD31.Ar/T.Ar measurements. Per-image comparison of manual and TWS-derived CD31.Ar/T.Ar measurements. CD31.Ar/T.Ar = CD31-positive area/tissue or ROI area (%).

Sample	Manual CD31.Ar/T.Ar (%)	TWS CD31.Ar/T.Ar (%)	Difference TWS–Manual
spine 1	6.25 ± 0.92	5.47 ± 0.08	−0.78
spine 2	8.38 ± 0.19	6.55 ± 0.07	−1.83
spine 3	11.07 ± 1.14	10.25 ± 0.02	−0.82
defect/regenerate 1	5.52 ± 0.27	4.77 ± 0.01	−0.75
defect/regenerate 2	7.26 ± 0.48	7.79 ± 0.21	+0.53
defect/regenerate 3	10.34 ± 0.53	11.19 ± 0.04	+0.85
femur 1	5.90 ± 1.64	4.48 ± 0.08	−1.42
femur 2	10.86 ± 0.44	8.21 ± 0.01	−2.65
femur 3	10.16 ± 0.44	7.68 ± 0.33	−2.48
tibia 1	2.84 ± 0.35	2.19 ± 0.09	−0.65
tibia 2	4.20 ± 1.01	1.43 ± 0.11	−2.77
tibia 3	4.67 ± 2.55	2.63 ± 0.01	−2.04

## Data Availability

The raw data supporting the conclusions of this article will be made available by the authors upon request.

## Supplementary Materials

The following supporting information can be downloaded at https://www.mdpi.com/article/10.3390/jimaging12090409/s1, Table S1: Decalcification and paraffin embedding workflow for rat bone samples; Figure S1: Representative counterstaining quality after CD31 immunohistochemistry; Protocol S1: Step-by-step Fiji/ImageJ workflow for CD31-positive area quantification; Figure S2: Representative examples of TWS-based segmentation used for quantitative CD31 analysis.

## Abbreviations

The following abbreviations are used in this manuscript:

°C	degrees Celsius
Ar.	area
ASBMR	American Society for Bone and Mineral Research
α-SMA	alpha-smooth muscle actin
CV	coefficient of variation
EDTA	ethylenediaminetetraacetic acid
min	minutes
PECAM-1	platelet endothelial cell adhesion molecule-1
ROI	region of interest
SD	standard deviation
TAr.	total area
TBS	tris-buffered saline
TWS	Trainable Weka Segmentation
μ	micrometers
