# Supplementary material for: Reproducible Semi-Automated Quantification of Vascularization in Bone Sections Using CD31 Immunohistochemistry and Trainable Weka Segmentation
Source: J Imaging. 2026 Sep 1;12(9):409. doi: 10.3390/jimaging12090409 (PMC13608717; doi:10.3390/jimaging12090409)
Supplement: Supplementary file 1 [file jimaging-12-00409-s001.zip › jimaging-4421494-supplementary.pdf]

Supplementary Table S1. Decalcification and paraffin embedding workflow for rat bone samples.

Step	Procedure	Reagent / Material	Duration / Conditions
1	Fixation	4% paraformaldehyde	24 h at 4 °C
2	Washing	PBS 1×, pH 7.2–7.4	6 × 5 min
3	Transfer to embedding cassettes	Labeled embedding cassettes	—
4	Decalcification	EDTA solution, pH 7.4	2–4 weeks, depending on sample size
5	Agitation / storage during decalcification	EDTA-containing vessels	Continuous gentle agitation; refrigerated storage
6	Post-decalcification rinse	Running tap water	1 h
7	Interim storage	70% ethanol	4–8 °C until processing
8	Device preparation	Tissue processor, paraffin embedding station, cooling plate (Leica Biosystems Nussloch GmbH, Nussloch, Germany)	Enable tissue processor ≥10 h before use
9	Dehydration I	70% ethanol	0.5 h
10	Dehydration II	70% ethanol	1 h
11	Dehydration III	80% ethanol	1.5 h
12	Dehydration IV	96% ethanol	1.5 h
13	Dehydration V–VII	100% ethanol	3 × 1.5 h
14	Clearing	Xylene	3 × 1.5 h
15	Paraffin infiltration	Paraffin, 58 °C	2 × 1.5 h
16	Embedding	Paraffin embedding station (HistoCore Arcadia, Leica Biosystems Nussloch GmbH, Nussloch, Germany)	Enable embedding station ≥5 h before use
17	Cooling	Cooling plate (Leica EG1150C, Leica Biosystems Nussloch GmbH, Nussloch, Germany)	Until complete solidification

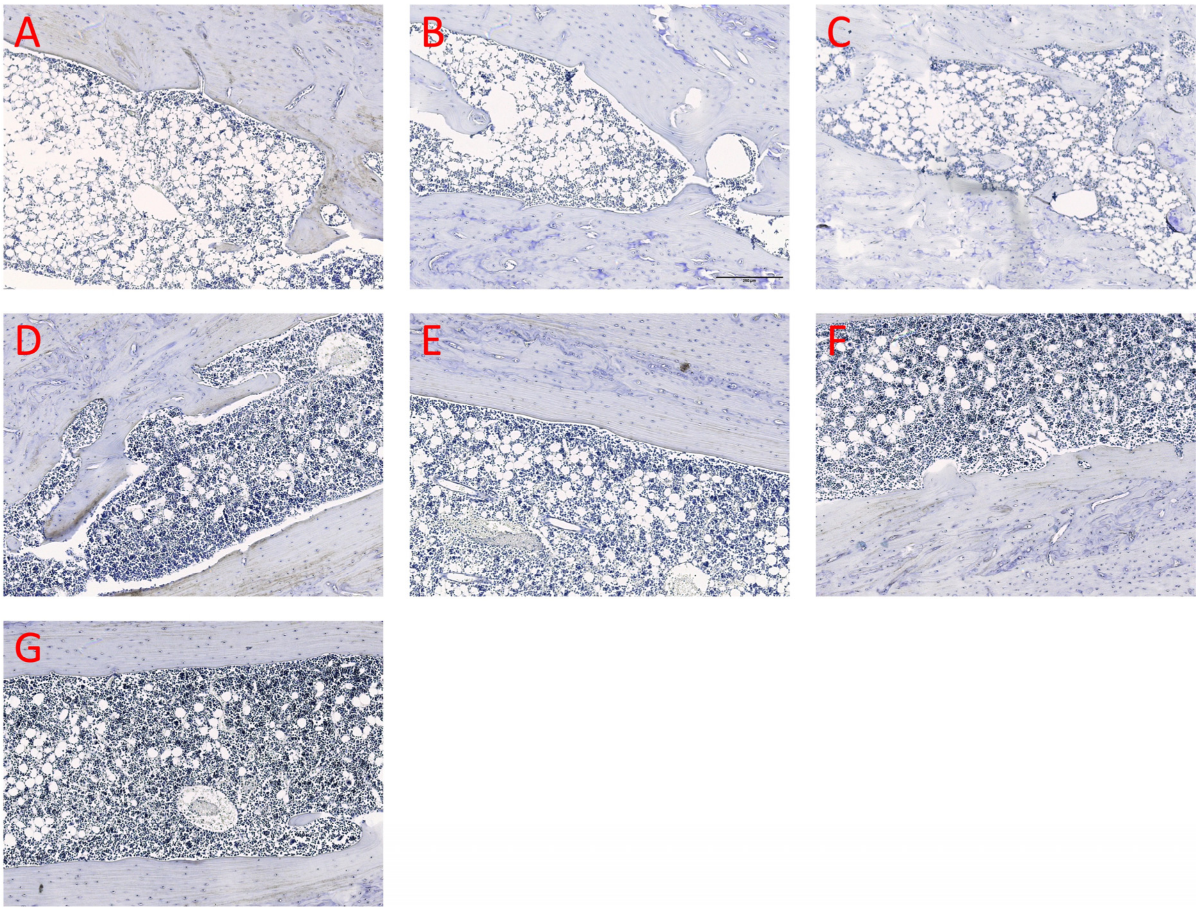

Supplementary Figure S1. Representative counterstaining quality after CD31 immunohistochemistry. Representative CD31-immunohistochemically stained bone sections counterstained with Instant Hematoxylin. Sections were counterstained for 10 seconds (A), 20 seconds (B), 30 seconds (C), 45 seconds (D), 60 seconds (E), 120 seconds (F) and 5 minutes (G). The counterstaining provided sufficient nuclear and tissue contrast while maintaining clear visual separation from the Vector Red CD31-positive signal.

Supplementary Protocol S1. Step-by-step Fiji/ImageJ workflow for CD31-positive area quantification

The corresponding analytical stages are illustrated in Figure 1 of the main manuscript

Image preparation and ROI definition

1. **Open the image in Fiji/ImageJ.**
   Drag and drop the microscopy image into Fiji/ImageJ or select **File → Open**.

2. **Define the region of interest (ROI).**
   Select the **Polygon Selection** or **Freehand Selection** tool from the Fiji/ImageJ toolbar and outline the predefined anatomical region to be analyzed.

3. **Save the ROI.**
   Open the ROI Manager via **Analyze → Tools → ROI Manager** and select **Add**. Save the ROI for consistent application during subsequent analysis.

4. **Remove areas outside the ROI.**
   Select **Edit → Clear Outside** to exclude image regions outside the predefined ROI.

5. **Optional: Crop the image.**
   Select **Image → Crop** if reduction of the image dimensions is desired. Cropping may reduce computational processing time but does not alter the predefined ROI.

Trainable Weka Segmentation

6. **Open Trainable Weka Segmentation.**
   Select **Plugins → Segmentation → Trainable Weka Segmentation**.

7. **Define the segmentation classes.**
   Create classes representing:

   - CD31-positive vascular signal,
   - counterstained/background tissue, empty background and/or relevant artifact-associated regions.

8. **Annotate representative training regions.**
   Using the freehand selection tool, mark representative areas for each class and assign them using **Add to class**. Include different staining intensities as well as representative transition regions between the red CD31-positive signal and blue hematoxylin-counterstained tissue.

9. **Train the classifier.**
   Select **Train classifier**.

10. **Inspect the segmentation result.**
    Visually compare the classifier prediction with the original image. If CD31-positive structures, weakly stained regions, or transition zones are not reliably classified, add further representative annotations and repeat **Train classifier**.

11. **Save the classifier.**
    Once satisfactory segmentation is achieved, select **Save classifier**. The saved classifier can

subsequently be applied to images acquired under comparable staining and imaging conditions.

Generation of the CD31-positive mask

12. **Generate the classified image.**
    Select **Create Result** in the Trainable Weka Segmentation window.

13. **Isolate the CD31-positive class.**
    Duplicate the classified result image (**Image → Duplicate**) and open **Image → Adjust → Threshold**. Select only the discrete pixel value assigned by TWS to the CD31-positive class.

14. **Generate the binary mask.**
    Convert the isolated CD31-positive class into a binary mask.

15. **Restrict the mask to the predefined ROI.**
    Reapply the saved ROI from the ROI Manager and use **Edit** → **Clear Outside** to ensure that only CD31-positive structures within the predefined ROI are quantified.

Quantitative analysis

16. **Measure the CD31-positive area.**
    Select **Analyze → Analyze Particles** and record the total CD31-positive area.

17. **Measure the ROI area.**
    Apply the saved ROI to the original calibrated image and determine its area using **Analyze → Measure**.

18. **Calculate the CD31-positive area fraction.**

CD31.Ar/T.Ar (%) = total CD31-positive area / ROI area × 100

19. **Quality control.**
    Overlay the detected CD31-positive regions onto the original histological image and visually inspect the segmentation before accepting the quantitative result.

Batch analysis

Once a classifier has been established and validated, subsequent images can be analyzed using the saved classifier. For larger datasets, the BeanShell script provided with this study can be used to automate the downstream analysis steps. The script is freely available at: https://github.com/Nick-Mattern/tws-area-makro.

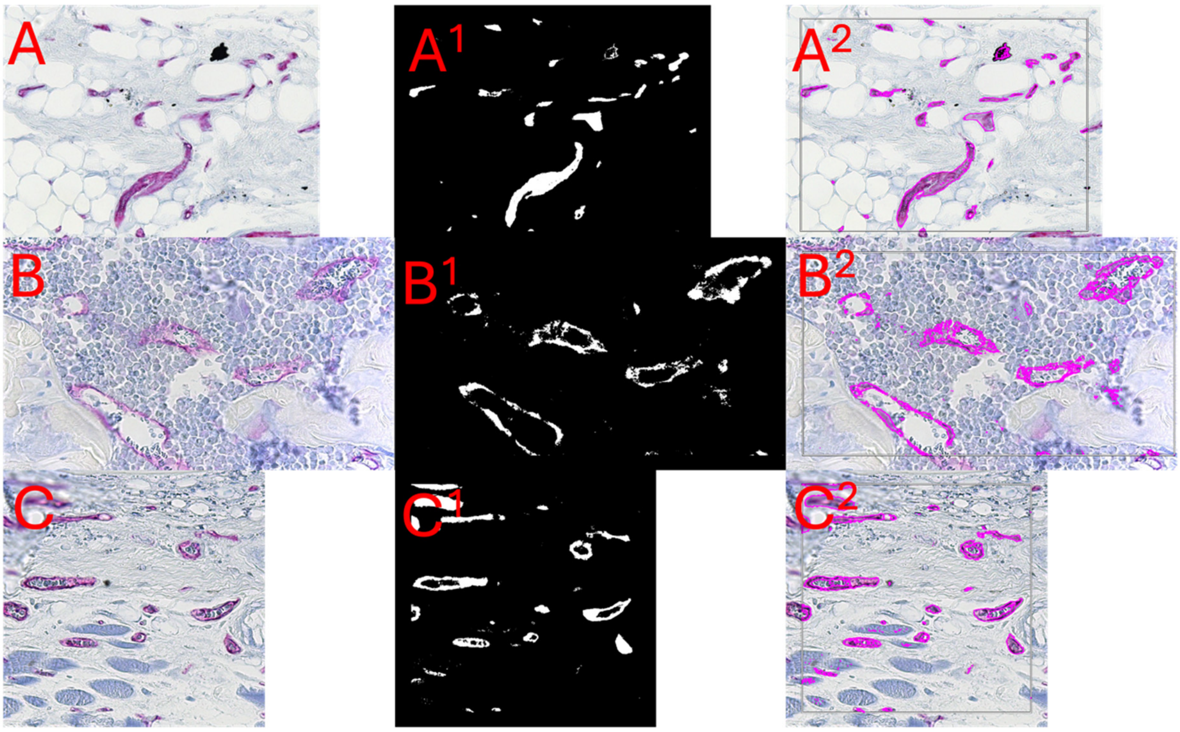

Supplementary Figure S2. Representative examples of TWS-based segmentation used for quantitative CD31 analysis. (A–C) Original, unprocessed CD31-immunohistochemically stained images from three representative samples included in the quantitative validation: (A) defect/regenerate 1, (B) spine 2, and (C) defect/regenerate 2. (A^1–C^1) Corresponding binary masks generated from the TWS-classified CD31-positive signal. White pixels represent regions classified as CD31-positive, whereas black pixels represent non-CD31-positive areas. (A^2–C^2) Overlays of the TWS-derived segmentation onto the corresponding original histological images, allowing direct visual assessment of the agreement between the detected regions and the underlying CD31-positive chromogenic signal. The faintly visible ROI boundaries in the overlay images indicate the predefined region used for quantitative analysis and are shown for orientation only. The examples illustrate the segmentation procedure underlying the quantitative CD31.Ar/T.Ar measurements reported in Table 3.
